# 

**Published:** 1899-08

**Authors:** 


### MARRIAGES.

On June 28th, Geo. C. Van Mater, M.D., D.V.S., graduate of American Veterinary College, class of 1886, to Miss Lillie V. Blinn, both of Brooklyn, N. Y.

### A. V. M. A.

### THIRTY SIXTH ANNUAL MEETING, AMERICAN VETERINARY MEDICAL ASSOCIATION.

*Headquarters.* The headquarters of the Association will be at the Hotel Manhattan, Forty-second Street and Madison Avenue, New York City, September 5, 6, and 7, 1899.

*Hotel Accommodations.* Within a radius of a few blocks from the meeting-place there are a number of other hotels, and numerous boarding and furnished-room houses.

It is the intention of the Committee of Arrangements to secure for the members of the Association and their families suitable accommodations. If they will communicate with Dr. H. D. Gill, Chairman of the local Committee of Arrangements, 154 East Fifty-seventh Street, New York City, stating full particulars as to the accommodations they may wish, all arrangements will be made in advance.

*Conjoined Veterinary Events:* 1. The American Veterinary Medical Association. 2. The New York State Veterinary Medical Society. 3. The New York County Veterinary Medical Association. 4. Association of Veterinary Faculties of North America. 5. Maine Veterinary Medical Association. 6. Experiment Station Veterinary Medical Association. 7. The Silver Jubilee of the Alumni Association of the American Veterinary College.

*Meetings.* Meetings will be held at the Academy of Medicine, 17–23 West Forty-third Street, between Fifth and Sixth Avenues. Hosack Hall, the most commodious in the building, has been engaged, together with a committee-room. The location is central, being within one block of headquarters, and five minutes' walk of the Grand Central Depot.

*Surgical Clinics.* Clinics will be held in the American Horse Exchange Building, Fiftieth Street and Broadway (within five minutes' walk of the meeting room), Wednesday and Thursday mornings, from 8 to 10 o'clock.

On account of the refrigerating facilities, the pathologic exhibit will be at Eastman's Abattoir, Fifty-ninth Street and Eleventh avenue (this is about fifteen minutes' walk from the meeting-room), Tuesday, 4 to 6 P.M.

Several instrument-makers have been invited to exhibit their stock. This will give veterinarians an opportunity to see all the latest surgical instruments and appliances.

*Entertainment.* The local Committee of Arrangements has made special provision for entertaining the ladies accompanying members and veterinarians.

Tuesday, September 5th, morning: Visit the Academy of Medicine to witness the opening exercises of the convention.

Afternoon: Carriage ride through Central Park and the Riverside Drive to Grant's Tomb.

Wednesday, September 6th, morning; Visiting large shopping districts under escort of local committee of ladies.

Evening: Theatre party.

Thursday, September 7th, morning: Visit to Museum of Art or Natural History.

Afternoon: Excursion and clam-bake at Rockaway Beach. This will consist of a sail through New York harbor, along the Long Island, Staten Island, and Jersey coasts, within full view of the Brooklyn Bridge, the Statue of Liberty, Governor's Island, and past the naval fortifications at Forts Hamilton, Wadsworth, and Lafayette, and the famous Coney Island. The steamer will return to New York in time for night trains.

The Association will be welcomed to Gotham at the Academy of Medicine by President James L. Robertson, of the County Association.

The banquet will take place on Wednesday evening, September 6th, at the Hotel Manhattan.

The following additional papers have been offered for this meeting:

Dr. H. D. Gill, New York, "Antitoxins."

Dr. W. Horace Hoskins, of Pennsylvania, "Some Aspects of Future Veterinary Legislation."

Dr. Wilfried Lellman, of New York, "Future Outlook of the Veterinary Profession."

Dr. Henry Henning, of New York, "Operation for Roaring."

Dr. W. L. Williams, of New York, "Etiology of Spavin and Its Allies."

Dr. A. T. Peters, of Nebraska. (Subject to be announced later.)

Dr. George B. Morse, of New York, "The Handling of Milk."

Dr. J. J. Repp, of Iowa. (Subject to be announced later.)

Dr. D. E. Salmon, of Washington. (Subject to be announced later.

*Surgical Clinics.*

Dr. W. L. Williams, of New York, "Caudal Myotomy," "Spaying a Mare or Bitch," "Administering Anæsthetics," "Exhibiting and Operating on an Improved Operating Table."

Dr. H. D. Gill, of New York, "Plantar Neurectomy."

Dr. George H. Berns, "Removal of Lateral Cartilage."

Dr. Wilfried Lellman, of New York. (Best operative case presenting itself in his practice at that time.)

Dr. S. J. J. Harger, of Pennsylvania, "Myoneurectomy."

Dr. L. A. Merillat, of Illinois. (Operation to be announced.)

Dr. J. M. Wright, or L. A. Merillat, of Illinois, "Operations on Teeth."

Dr. Henry Henning, of New York, "Operation for Roaring."

Dr. William Sheppard, of New York. (Best operative case presenting itself in his practice at that time.)

Dr. J. H. Wattles, of the Western Veterinary College, of Kansas City, Mo., will be among the visitors to the convention.

Dr. W. H. Kelly, of Albany, N. Y., will present a practical paper considering the uses, etc., of fluid extracts made with acetic acid, recently placed upon the market by one of the foremost drug-firms of this country.

Secretary Ridge, of Pennsylvania, in a strong circular, sums up four cogent reasons why our membership should be enlarged. We heartily second the appeal to all JOURNAL readers over the land, and urge their consideration by every reader not a member of the association.

1st. It is an honor to belong to the highest veterinary medical association in this country, and all veterinarians should have a desire to add their names to its roll; it gives them a standing that justly belongs to every good veterinarian.

2d. It is a source of education that cannot be received by any other method. We get the culture of many minds concentrated and meted out to us in a manner most helpful and which would not reach us through other channels.

3d. We should understand what will do the most good to the greatest number in our profession; by uniting we can do this; by individual action we cannot. To quote Professor Hamilton, in speaking of the farmers' institute, he says: "The great weakness of our country people lies in their lack of consolidation of thought upon a given question. When all of the country people agree upon any given subject, their desires will be gratified. Their failure lies in the lack of agreement, and the lack of agreement is usually due to a lack of accurate knowledge of the subject, and its true bearing upon their industry."

This, we think is applicable to us as a profession. While local associations are good educators, the meeting of the whole body of veterinarians can consolidate thought upon questions that are of vital importance to us as American veterinarians, that cannot be done by local or other associations.

4th. The mercenary view—take it in a business light—will it pay? Yes, it will pay, even if you never attend any meetings. You will receive the printed proceedings of the association, and in numerous other ways receive more than your money's worth.

I trust that you will find the above reasons valid, and that you will write to me for an application blank, and a copy of the constitution and by-laws, which I will take pleasure in sending you by return mail, and if you decide to apply for membership—and I sincerely hope you will—I will be pleased to endorse your application and forward it to the secretary of the association.

Among those who expect to be present at the New York gathering from the city of Cincinnati may be mentioned Drs. J. C. Meyer, Louis P. Cook, and William G. Shaw. The latter two are representatives of the Bureau of Animal Industry.

### ASSOCIATION OF VETERINARY FACULTIES AND EXAMINING BOARDS OF NORTH AMERICA.

The sixth annual meeting of this organization will be held on September 7th, when the following papers will be presented:

"Education of the Veterinarian," Dr. James Law.

"Objects and Aims of an Association of Faculties," Dr. C. Barnwell Robinson.

"The Teaching of Practical Surgery," Dr. W. L. Williams.

"Clinical Lectures," Dr. S. J. J. Harger.

"State Examinations," Dr. A. W. Clement.

Other papers will be announced at the meeting.

### THE UNITED STATES EXPERIMENT STATION VETERINARY MEDICAL ASSOCIATION.

Third annual meeting, Hotel Manhattan, Monday evening, September 4th: President, Dr. James Law, Ithaca, N. Y.; Vice President, Dr. J. W. Connoway, Columbia, Mo.; Secretary, Dr. A. T. Peters, Lincoln, Neb.; Executive Committee, Drs. M. Stalker, A. W. Bitting, M. H. Reynolds. Programme: 8.00 P.M., annual meeting convenes; association business. The following papers will be read:

"Failure of the Tuberculin Test," Dr. F. L. Russell, Maine.

"Cerebro-Spinal Meningitis in Horses," Dr. S. S. Buckley, Maryland.

"Blackleg and Blackleg Vaccine." Dr. Paul Fischer, Kansas.

"Glanders and Its Possible Cure," Dr. W. C. Langdon, North Dakota.

"Laboratory Suggestions," Dr. C. A. Cary, Alabama.

### ALUMNI ASSOCIATION—SILVER ANNIVERSARY OF THE AMERICAN VETERINARY COLLEGE.

This important event will take place on the evening of the 5th of September. The reception of Professor A. Liautard, dean and organizer of the College, will, no doubt, prove one of those rare occasions in the history of the profession in a new country that will long be remembered among the important epochs. There will be a re-union of graduates, many of whom will return to their college parent for the first time since their graduation, and thus add and receive pleasure and delight. The committee in charge are making extensive provisions for a great outpouring of the A. V. C. boys.

---

THE MAINE STATE VETERINARY MEDICAL ASSOCIATION will hold a special meeting in New York, on September 5th, in conjunction with the national organization.

### PERSONALS.

Dr. T. J. Turner, of the Bureau of Animal Industry, late inspector in charge at Indianapolis, Ind., has been transferred to Louisville, Ky.

Dr. J. W. Connoway, of Missouri, has an article on the results of inoculation experiments for Texas fever and the development of immune animals in the July 19th issue of the *Breeders' Gazette*.

Dr. Charles S. Gelbert, of the class of '99, Veterinary Department of the University of Pennsylvania, has located in Scranton, Pa.

Dr. H. E. Ryder, of Buffalo, N. Y., has been added to the force of the Bureau of Animal Industry at Indianapolis, Ind., as an assistant inspector.

Dr. Wm. Dougherty, of Baltimore, spent two weeks in the latter part of July at Saratoga, Boston, and other Eastern points.

Dr. Geo. W. Butler, of Circleville, Ohio, has accepted an assistant inspectorship in the Bureau of Animal Industry at Milwaukee, Wis.

Dr. R. S. Huidekoper spent a portion of the summer season at Kirkland, Pa., in the vicinity of Philadelphia.

Dr. Sorrensen, of the Bureau of Animal Industry, recently of Louisville, Ky., now has charge of the work at Indianapolis.

Dr. Lloyd Zaner, formerly of Stillwater, has located at Wilkesbarre, Pa. Dr. Zaner graduated at the Veterinary Department of the University of Pennsylvania, class of '98.

Dr. Dolan, of the Bureau of Animal Industry, of Indianapolis, Ind., is enjoying a month's leave of absence, visiting his home near Boston, Mass.

Dr. E. J. Carter, of Pittsburg, who retired from active practice for several years to enter the field of proprietary medicine, has announced by letter his purpose of again resuming private practice in the "Smoky City."

Dr. Tait Butler, of Kansas City, has accepted a position as assistant inspector in the Bureau of Animal Industry at Indianapolis, Ind.

Dr. Leonard Pearson, of Philadelphia, was summoned to Boston in July to attend the burial services of a nephew.

Dr. O. C. Farley, of New York City, will officiate as veterinarian to the Newport horseshow in September.

Dr. J. S. Cattanach, of New York City, filled the rôle of veterinarian to the Atlantic City horseshow in July.

Dr. L. L. Cheney, of the class of '99, Veterinary Department of the University of Pennsylvania, has located at Augusta, Ga., and reports the field as promising.

Dr. Thomas Castor, of the Bureau of Animal Industry at Buffalo, N. Y., spent July 4th at his home in the Quaker City, visiting parents and friends. These trips, occurring with increasing regularity, excite suspicion in the minds of careful observers—probably other items later on.

Dr. P. K. Jones, of Pittsburg, associated in practice with City Veterinarian McNeil, has fitted up a canine infirmary in connection with their equine hospital.

Prof. A. Liautard has translated into English for the Seventh International Veterinary Congress, to be held in Baden-Baden in August, several of the most important papers, to facilitate the better discussion of these subject.

Drs. Pearson, Marshall, and Hoskins, representing the veterinarians of Philadelphia, were visitors to the Mayor's office in July in furtherance of plans looking to improvements in the meat-inspection and milk-inspection service.

State Secretary Ridge, of Pennsylvania, is out in a strong circular, urging increased interest and attendance upon the meeting in New York in September.

Dr. Wm. H. Pendry, of Brooklyn, N. Y., met with a slight accident in July while casting one of his patients.

Dr. R. H. Thomas, formerly of Shelbyville, Tenn., is now a resident of Rome, Ga.

Dr. H. Jensen, formerly of Jewell, Iowa, is now a resident of Weeping Water, Neb., and conducts a drugstore in connection with his practice.

Dr. W. Horace Hoskins, of Philadelphia, was a visitor to Gotham in July, incidental to the coming silver anniversary of the American Veterinary College in September.

Dr. J. H. Gain, of Rushville, Neb., has again returned to the ranks of the profession as a practitioner, having given up the commercial occupation he was engaged in.

The JOURNAL inadvertently stated in its June number the change of place of Dr. J. H. McNeal from Buffalo to Brooklyn. Dr. McNeal will remain at Buffalo for the present.

Secretary Rhoads, of the Pennsylvania State Veterinary Association, promises an excellent programme for the meeting at Scranton in September.

Dr. A T. Peters, of Lincoln, Neb., has been in impaired health for some time, but hopes to make the trip to New York in September.

Prof. H. A. Morgan, entomologist to the Louisiana State Experiment Station, has just issued one of the most completely illustrated bulletins on "Ticks and Texas Fever," which will prove of great importance and benefit to the livestock raisers of the South and the livestock commercial interests of the North.

Dr. Frederick Taylor, class of '99, Veterinary Department of the University of Pennsylvania, has located at Sewickley, Pa. Dr. N. L. Townsend, of the same class, has opened an office at Moorestown, N. J.

Dr. Leonard Pearson, State Veterinarian of Pennsylvania, has been summoned before the Commission of the New York State Legislature to investigate the subject of tuberculosis in cattle, looking to future legislation of a more extended character

C. R. Perkins, D.V.M., has been appointed as assistant in clinical surgery at the New York State Veterinary College for the year 1899 to 1900.

